# Role of Extracellular Vesicles as Mediators of Cell Communication and Novel Biomarkers in Sepsis

**DOI:** 10.3390/jcm14186649

**Published:** 2025-09-21

**Authors:** Alberto Repici, Giovanna Piraino, Vivian Wolfe, Jennifer Kaplan, Takahisa Nakamura, Basilia Zingarelli

**Affiliations:** 1 Division of Critical Care Medicine, Cincinnati Children’s Hospital Medical Center, Cincinnati, OH 45229, USA; alberto.repici@cchmc.org (A.R.); giovanna.piraino@cchmc.org (G.P.); vivian.xue@cchmc.org (V.W.); jennifer.kaplan@cchmc.org (J.K.); 2 Department of Chemical, Biological, Pharmaceutical and Environmental Sciences, University of Messina, 98122 Messina, Italy; 3 Department of Pediatrics, College of Medicine, University of Cincinnati, Cincinnati, OH 45221, USA; 4 Division of Diabetes and Endocrinology, Cincinnati Children’s Hospital Medical Center, Cincinnati, OH 45229, USA; takahisa.nakamura@cchmc.org

**Keywords:** extracellular vesicles, sepsis, organ failure, cell communication

## Abstract

Small extracellular vesicles (sEVs), typically <200 nm in diameter, have emerged as key mediators of intercellular communication, transferring bioactive molecules such as proteins, lipids, and nucleic acids between cells. This review focuses on the growing significance of sEVs in the context of sepsis, a life-threatening syndrome caused by a dysregulated immune response to infection. Sepsis remains a major global health challenge due to its complex pathophysiology, rapid progression, and the limitations of current diagnostic tools, which often fail to detect the condition early or accurately assess the host’s immune status. As interest grows in precision diagnostics, sEVs have gained attention for their potential as biomarkers in liquid biopsy—a minimally invasive approach that analyzes circulating vesicles to monitor disease. Small EVs reflect the physiological state of their cells of origin and can provide real-time insights into immune activation, inflammation, and pathogen presence. This review explores the mechanisms by which sEVs contribute to immune modulation in sepsis, recent advances in understanding their biogenesis and uptake, and their diagnostic and prognostic potential. By highlighting the role of sEVs in sepsis, we aim to underscore their promise in improving early detection, guiding therapeutic decisions, and advancing personalized medicine.

## 1. Introduction

Sepsis is a life-threatening condition that arises when the body’s response to an infection causes organ dysfunction. Sepsis is determined by intricate interactions among the infecting organism, the host immune system, inflammatory responses, and coagulation [1]. Sepsis is triggered primarily by two types of molecules: pathogen-associated molecular patterns (PAMPs) and damage-associated molecular patterns (DAMPs). PAMPs are substances produced by microorganisms, such as lipopolysaccharides (LPS), bacterial glycans, lipoteichoic acid, bacterial DNA and viral RNA, while DAMPs are released by stressed or dying cells. When PAMPs and DAMPs bind to pattern recognition receptors (PRRs), they activate an inflammatory response [2]. The systemic inflammatory response triggered by sepsis may result in multi-organ dysfunction, which is the primary cause of mortality in sepsis.

Extracellular vesicles (EVs) are membrane-bound nanoparticles that are released by cells throughout the body [3]. All eukaryotic cells and some prokaryotic cells have the ability to release these EVs into the surrounding milieu [4]. Proteins, lipids, RNA, and DNA are the bioactive compounds found within the phospholipid double layer matrix, and they reflect the biological characteristics of the cell of origin. Cryo-electron microscopy technique, which is currently the only method that allows direct visualization of EV ultrastructural characteristics [5], reveals that EVs typically have a spherical or ovoid shape, mainly determined by their lipid double layer structure composed of cholesterol, sphingolipid, ceramide and diacylglycerol [6]. EVs can be classified based on their size, which is normally between 0 nm and 5 μm, their biogenesis, and their composition [7]. According to these characteristics, EVs can be broadly categorized into exosomes, microvesicles, and apoptotic bodies (Table 1). Exosomes have a diameter between 40 and 120 nm and represent the smallest EVs produced by the process of exocytosis by multivesicular endosomes [8]. Microvesicles are between 100 and 1000 nm in size and are the result of outward budding and fission of the plasma membrane [9]. Apoptotic bodies are the biggest EVs with a diameter of 1000–5000 nm and are the byproducts of the cellular apoptotic process [10]. Recently, the International Society for Extracellular Vesicles ISEV) recommended using size-based terminology according to the Minimal Information for Studies of Extracellular Vesicles (MISEV) guidelines, to refer to small EVs (sEVs) as particles usually smaller than 200 nm, and large EVs (lEVs) if their size exceeds 200 nm, according to specific separation methods [11]. This terminology has become necessary since it is difficult to identify different EVs due to overlapping characteristics. Therefore, in this review, we will adopt this terminology according to the size of the particles described in the original articles.

Since almost every cell in our organism has the capacity to produce sEVs, the membrane composition of the sEVs can vary greatly. As a consequence, the membrane will have properties that are comparable to those of the parental cell from which it originated [21]. The characteristics that sEVs inherit from the parental cell may also include surface proteins with the potential to interact with their environment [22]. The cargo composition of sEVs may also be influenced by the original tissue type [23]. Small EVs encapsulate a diverse array of bioactive molecules, such as RNAs, proteins, and lipids, which are functionally relevant for cell–cell communication. Since sEVs may be found in practically every sample of bodily fluid, including blood, saliva, cerebrospinal fluid, and urine, their biological inheritance makes them good biomarkers for the assessment and early identification of potential disorders [24]. In this review, we will examine and discuss the remarkable complexity of sEVs and the most recent advancements in the applications of sEVs not only for diagnostic applications but also as potential carriers in drug delivery. Our literature search strategy included the use of keywords and focused primarily on recent publications. Seminal papers were also included to provide historical context and foundational insights.

## 2. Biogenesis and Functional Cargo of sEVs

**Figure 1:**
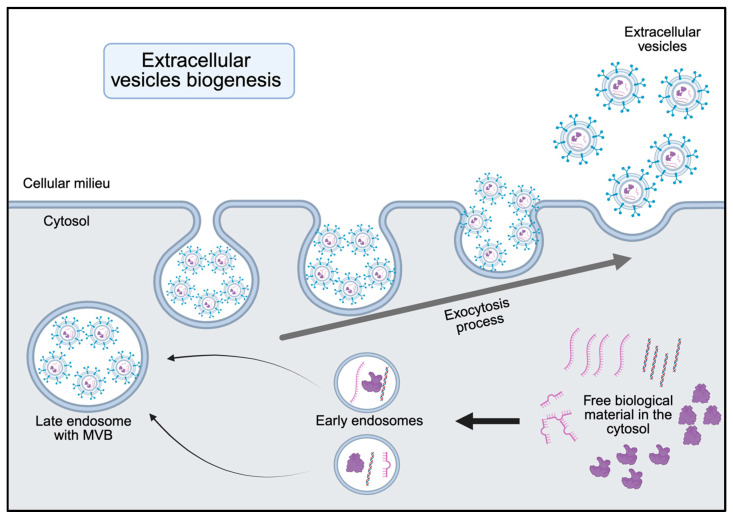
Small extracellular vesicles biogenesis. This schematic illustrates the cellular pathways involved in the formation of small extracellular vesicles (sEVs). The process begins with endocytosis, leading to the formation of early endosomes, which mature into late endosomes. During this maturation, inward budding of the endosomal membrane leads to the formation of multivesicular bodies (MVBs). During the formation of these inward buds the endosome incorporates specific proteins, lipids, and nucleic acids from the surrounding cytosol or other organelles. Upon fusion of MVBs with the plasma membrane, sEVs are released into the extracellular space.

Small EVs biogenesis involves a highly regulated, multi-stage process that begins with the invagination of the cell plasma membrane internalizing cellular molecules and neighboring foreign materials into small vesicles known as early endosomes. These early endosomes are intracellular compartments where internalized material is initially processed. The maturation of early endosomes implies that the recently internalized material may undergo degradation or be expelled from the cell if considered harmful, and the early endosomes subsequently mature to become late endosomes, which contain intraluminal vesicles. These intraluminal vesicles are formed by inward budding of the endosomal membrane and represent the characteristic feature of mature late endosomes, also known as multivesicular bodies (MVBs). MVBs may either be secreted from the cell via an exocytosis process, where they first fuse with the plasma membrane before dispersing into the intercellular space, or they can be degraded by lysosomes (Figure 1).

The small EVs are usually carriers of non-coding RNAs, including long non-coding RNAs (lncRNAs), circular RNAs (circRNAs), and microRNAs (miRNAs) [25]. Small EVs may also carry a wide range of proteins (transmembrane and non-membrane), which modulate complex processes of cell interaction. Proteins most abundantly present are typically tetraspanins (such as CD9, CD63, CD81, and CD82), which play a key role in the processes of vesicle trafficking and membrane fusion with the host cell [26]. Adhesion proteins (ICAMs) recognize the molecular targets to which sEVs bind [27]. Rab27a and Rab27b, which belong to the Rab GTPases family, regulate the complex network of cellular interactions [28]. Alix and TSG101 are associated with the Endosomal Sorting Complex Required for Transport (ESCRT), which modulates MVB maturation and development [29,30]. Small EVs may also carry heat shock proteins (HSPs) [31]; which are involved in antigen exposure, and major histocompatibility complex class I and II (MHC-I/II), responsible for the presentation of the antigens to T cells [32].

## 3. Function in Cell–Cell Communication

Following their biogenesis, sEVs play their role as molecular messengers through autocrine, paracrine, or endocrine actions, by actively joining in cell–cell communication [33]. The autocrine role of extracellular vesicles refers to the ability of cells to influence their own behavior through the release and subsequent interaction with their own vesicles. In addition, intercellular trafficking of sEVs can take place with distant cells as a form of endocrine signaling or with nearby cells as a form of paracrine signaling. The molecular information contained in sEVs, which reflects the phenotypic characteristics of their cell of origin, serves as a message delivered to recipient cells. Unlike soluble molecules such as hormones and cytokines, which diffuse in a non-specific manner, sEVs exhibit a degree of specificity, recognizing and targeting particular cells [34]. To understand how cell-to-cell communication via sEVs functions, it is essential to examine certain characteristics of these vesicles, including their cell selectivity, the nature of their binding interactions, and the intracellular trafficking that occurs once the sEVs are internalized by the recipient cell [35].

Small EVs carry functional surface receptors derived from their parent cells [36]. Upon interaction with recipient cells, these vesicle-associated receptors can be activated either directly by ligand binding on the target cell membrane or through conformational changes during membrane fusion. Once activated, these receptors may trigger specific intracellular signaling cascades, mimicking or modulating physiological processes typically initiated by endogenous membrane proteins. This receptor-mediated mechanism allows sEVs to not only deliver passive cargo but also to actively modulate the signaling landscape of the recipient cell, influencing pathways involved in cell proliferation, differentiation, immune modulation, and survival. Glycoproteins, lipids, glycans, and tetraspanins are examples of surface components that are essential for regulating the first contact and recognition between sEVs and target cells.

Depending on the molecular composition of the target cell membrane and the vesicle, these molecules function as specific interaction mediators that can promote binding and also affect the selectivity of sEVs uptake [37]. For example, integrins are essential for interactions between cells and sEVs, especially when cellular invasion is involved. Certain integrins, such as α6β4 and α6β1, have been associated with lung metastasis, whereas integrin αvβ5 is linked to liver metastasis, according to proteomic analysis of sEVs [38]. The membrane of the recipient cell also expresses several types of receptors that the sEVs can engage with, including transmembrane proteins [39]. Phosphatidylserine (PS), a glycerophospholipid present on the membrane of sEVs, functions as a key signaling and recognition molecule in cell communication. Its interaction with the Tim4 receptor is calcium-dependent and represents one of the most well-characterized mechanisms of sEVs uptake [40].

**Figure 2:**
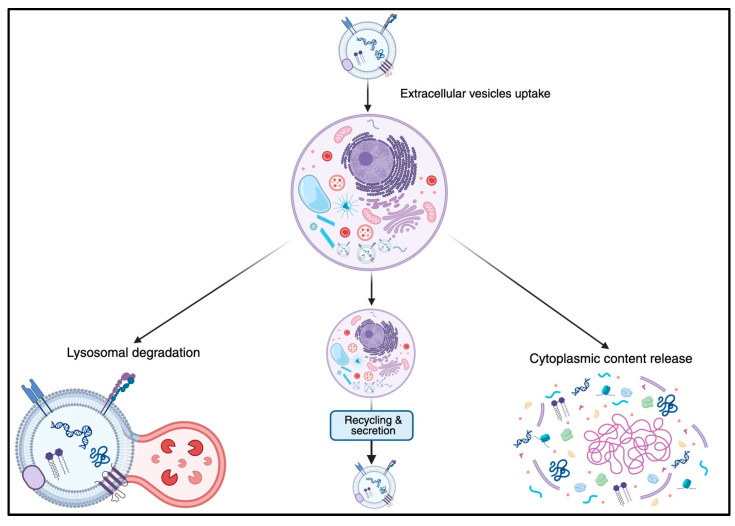
Intracellular traffic of the Small Extracellular Vesicles. This schematic illustrates the fate of small extracellular vesicles (sEVs) following their uptake into the cell. After internalization, sEVs may fuse with lysosomes and undergo lysosomal degradation, which leads to the breakdown of EVs and their cargo. Small EVs or their components may be sorted into recycling endosomes and returned to the plasma membrane for reuse or re-secretion. In some instances, sEVs may release their cargo directly into the cytosol through membrane fusion or disruption, influencing intracellular signaling and gene regulation.

Once bound to the cell surface, the sEVs can go through an uptake mechanism and undergo complex intracellular trafficking, which follows one of three main pathways: lysosomal degradation, recycling and secretion, or cytoplasmic content release [41] (Figure 2). The most common route is lysosomal degradation, where the vesicles and their contents are digested. Alternatively, some sEVs are recycled and released back into the extracellular space, contributing to the modulation of the surrounding environment. In other cases, sEVs release their cargo directly into the cytoplasm, a process that can directly influence the physiological functions of the recipient cell [41].

Small EVs have a relatively short half-life. Lai et al. engineered EVs with a membrane-bound Gaussia luciferase and tracked their presence in blood at multiple time points after intravenous injection in mice. By quantifying bioluminescence over time and applying a two-phase exponential decay model, they found that the engineered-sEVs underwent a rapid distribution phase with a half-life of ~20 min, followed by a slower elimination phase with a half-life of ~185 min. Most of the sEVs were cleared from circulation within 1–2 h, primarily via hepatic and renal routes [42]. Importantly, sEVs do not only transport proteins but also carry nucleic acids such as DNA, RNA, mRNAs, and miRNAs [43]. These molecules, once released into the cytoplasm of the recipient cell, have the potential to induce both genetic and epigenetic modifications. Through this mechanism, sEVs can regulate gene expression, influence intracellular signaling pathways, and even reprogram the functional identity of the target cell. This functional duality—acting both as carriers of nucleic acids and initiators of signaling through receptors—highlights the complexity and precision of vesicle-mediated communication, distinguishing sEVs from other extracellular signaling molecules [44].

## 4. Role of sEVs in Immune and Inflammatory Responses

Because of their cargo, sEVs affect the behavior or differentiation of nearby immune-competent cells. For example, dendritic cells can develop into various immune system cells with distinct functions after interacting with sEVs [45]. Small EVs actively participate in antigen presentation by different modalities, contributing to the maturation of immune cells, especially during several clinical conditions. Small EVs from human adipose-derived stem cells have the ability to affect macrophage and dendritic cell polarization, therefore fostering an equilibrium between M1 (pro-inflammatory) and M2 (anti-inflammatory) responses [46]. For example, several miRNAs carried by sEVs, such as miR-221 and miR-374b-5p, derived from mouse mammary epithelial cells, and miR-30d-5p, derived from mouse neutrophils, have been associated with the promotion of the M1 macrophage phenotype and the exacerbation of inflammatory responses [47,48,49]. In a murine model of sepsis, miR-155 carried by serum-derived sEVs promotes M1 macrophage polarization by activating the nuclear factor kappa-light-chain-enhancer of activated B cells (NF-κB) signaling pathway and inhibiting Src homology 2 domain-containing inositol-5-phosphatase 1 (SHIP1) and suppressor of cytokine signaling 1 (SOCS1), thereby enhancing macrophage proliferation and amplifying inflammation. MiR-155 also aggravates inflammatory responses in the central nervous system by stimulating the activation of microglia and astrocytes [49].

**Figure 3:**
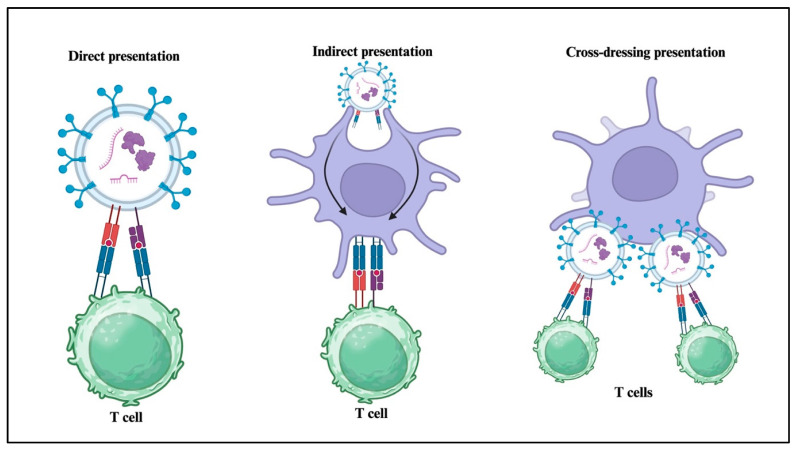
Antigen presentation facilitated by Small Extracellular Vesicles. This schematic illustrates the diverse mechanisms by which small extracellular vesicles (sEVs) contribute to antigen presentation. *Direct presentation:* Small EVs bearing surface MHC-I or MHC-II molecules loaded with antigenic peptides can directly stimulate T cells. *Indirect presentation:* Small EVs transfer antigens to professional antigen-presenting cells (APCs), such as dendritic cells, which process and present the antigens via their own MHC molecules. *Cross-dressing:* APCs acquire intact MHC-peptide complexes from small EVs without processing, enabling rapid presentation to T cells.

Small particles released by Dendritic Cells (DCs) also play a pivotal role in immune regulation due to their intrinsic properties and their natural ability to transport immunogenic molecules, including MHC class I and II, co-stimulatory proteins, adhesion molecules, and tetraspanins [50]. The sEVs contribute to immune system function through three main processes. First, in direct presentation, sEVs from APCs like DCs directly interact with T cells using MHC-peptide complexes and signaling molecules. Second, in indirect presentation, sEVs transfer their antigenic peptides to other APCs, which then present these peptides to T cells. Lastly, in cross-dressing, sEVs attach to the surface of APCs, allowing them to present their MHC-peptide complexes directly to T cells, while the APCs provide the necessary activation signals [51] (Figure 3). DC-derived sEVs can directly activate memory CD4^+^ and CD8^+^ T lymphocytes, whereas the activation of naive T cells requires the uptake of sEVs by other DCs, which can present MHC-peptide complexes without directly expressing these molecules [52].

Additionally, DC-derived sEVs contribute to the maturation of other immune cells and promote the differentiation of T lymphocytes into specific subtypes, including Th1, Th2, and Treg cells [53]. The sEVs also play a direct role in modulating T lymphocyte responses by delivering specific antigens capable of activating both T and B lymphocytes, thereby promoting the establishment of immunological memory. They can present antigens to T lymphocytes more efficiently than classical mechanisms, without requiring further antigen processing by active presenting cells (APCs) [54]. In the absence of APCs, sEVs expressing molecules such as ICAM-1 and B7 co-stimulatory proteins can induce the proliferation of CD8^+^ T lymphocytes and promote their differentiation into effector cells [55]. These particles can also activate DCs through the cyclic GMP-AMP synthase/stimulator of interferon genes (cGAS/STING) pathway and the activation of interferon regulatory factor 3 (IRF3)-regulated genes, thereby enhancing the antiviral immune response [56,57].

Parental cells exhibit the capacity to finely modulate the molecular cargo of sEVs, enabling the selective enrichment of specific functional biomolecules. Cytokines play a key role in the pathogenesis of sepsis; their action is described as a “cytokine storm” [58], and sEVs emerge as important vehicles for the transport of these molecules. Small EVs not only carry these cytokines but can render them biologically active when they interact with the target cells. The content of sEVs, therefore, has been analyzed to gain information about the inflammatory and immune processes taking place during sepsis. For example, in a mouse sepsis model, sEVs have been shown to contain pro-inflammatory cytokines such as Interleukin (IL)-1β, IL-2, IL-6, and TNF-α at early stages, followed by IL-12, IL-15, IL-17, and IFN-γ at late stages [58]. Similarly, the anti-inflammatory cytokines IL-4 and IL-10 present in sEVs increase during the late stage of sepsis [59].

In vitro studies have also confirmed that sEVs released by murine RAW264.7 macrophages infected with bacteria exert pro-inflammatory effects [60]. These observations have prompted the concept that blocking EV biogenesis could attenuate the systemic inflammatory response and protect against organ dysfunction. For example, pretreatment of RAW264.7 macrophages with GW4869, a known blocker of EV generation and release, significantly reduced endotoxin-induced release of sEVs and the production of the pro-inflammatory cytokines TNF-α, IL-1β, and IL-6. Similarly, in vivo studies in mice subjected to endotoxin shock or experimental sepsis demonstrated that pretreatment with GW4869 significantly lowered serum levels of sEVs and inflammatory cytokines, reduced cardiac inflammation, attenuated myocardial depression, and improved survival [60]. These findings suggest that pharmacological inhibition of EV release, such as with GW4869, may represent a promising therapeutic strategy to limit systemic inflammation, preserve cardiac function, and improve clinical outcomes in sepsis [60].

One of the complications that can arise following sepsis is the development of acute respiratory distress syndrome (ARDS). In this context, the sEVs may act as functional vectors that reflect the progression of sepsis. In a clinical study in adult patients [61] circulating sEVs from immune, endothelial, and platelet cells during sepsis reflected cellular activation states and carried bioactive molecules such as caspase-1 and miR-126, directly influencing target cell function. In this study, the patients with circulating sEVs that induced cellular injury and had increased caspase-1 activity were significantly more likely to develop ARDS. Moreover, levels of miR-126-3p in endothelial cell-derived sEVs were significantly reduced in ARDS patients compared to healthy controls [61].

Additional experimental evidence supports the importance of sEVs content in the modulation of sepsis progression. In a model of murine polymicrobial sepsis [62], injection of mesenchymal stem cells (MSCs) lacking miRNA-223 (miR-223-KO) failed to protect the hearts of septic animals, in contrast to wild-type (WT) MSCs, whose protective effects were attributed to the release of sEVs containing miR-223. At molecular analysis, sEVs derived from miR-223-KO MSCs exacerbated cardiac injury by inducing the expression of pro-inflammatory proteins such as Semaphorin 3A and Signal Transducer and Activator of Transcription (STAT)-3. Conversely, in vivo treatment with WT sEVs enriched in miR-223 suppressed these proteins in cardiomyocytes, thereby reducing inflammation and apoptosis. These findings demonstrate that sEVs miR-223 is essential for MSC-mediated cardio-protection in sepsis, further reinforcing the concept that specific miRNAs within sEVs are key components in the progression of sepsis-related complications and may represent therapeutic targets [62].

It must be noted that selected sEVs populations may have also remarkable anti-inflammatory and reparative effects and these properties have been explored for their therapeutic potential in experimental mouse models of sepsis. For instance, endothelial progenitor cell–derived sEVs delivered in mice subjected to sepsis significantly improved survival, reduced microvascular leakage, and attenuated multi-organ injury. These effects were associated with specific miR-126-3p and 5p in the cargo which suppressed endothelial High Mobility Group Box 1 (HMGB1) and Vascular Cell Adhesion Molecule 1 expression and preserved vascular integrity [63]. MSC–derived sEVs have also shown anti-inflammatory activity and have mitigated organ injury in rodent sepsis models [64,65]. This dual pro-inflammatory and anti-inflammatory role highlights the potential of sEVs as diagnostic markers and therapeutic targets for managing sepsis-induced organ dysfunction.

## 5. Role of sEVs in Coagulation and Coagulopathy in Sepsis

**Figure 4:**
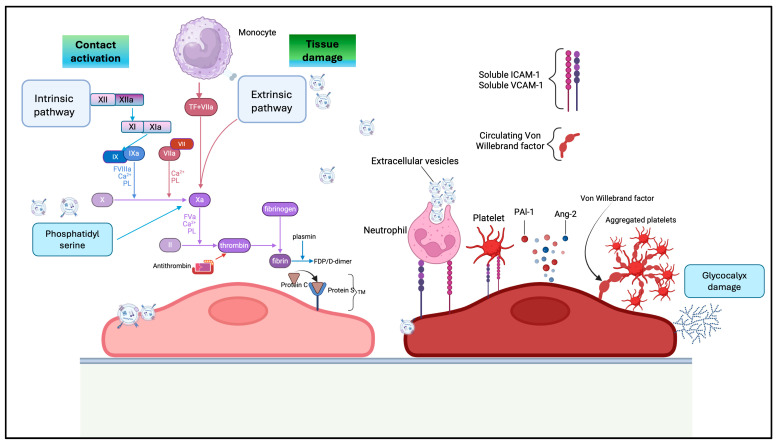
Small extracellular vesicles (sEVs) play a multifaceted role in the regulation of coagulation. Their effects can be categorized into two main pathways: direct and indirect. *Direct pathway:* sEVs can directly interact with coagulation factors at various stages of the coagulation cascade. For instance, they may present phosphatidylserine on their surface, which provides a catalytic platform for the assembly of coagulation complexes. Additionally, some sEVs carry tissue factor, a key initiator of the extrinsic pathway, thereby directly promoting thrombin generation and fibrin formation. *Indirect pathway:* Small sEVs can also indirectly influence coagulation by reprogramming the behavior of cells within the vascular and immune environments. They can modulate the phenotype of endothelial cells, monocytes, and platelets, potentially enhancing their procoagulant activity or altering their response to injury or inflammation. This cellular reprogramming can shift the hemostatic balance toward a prothrombotic or antithrombotic state, depending on the context.

Small EVs contribute simultaneously to cellular homeostasis and haemostasias, due to the presence of various membrane receptors that influence the surrounding environment. Circulating sEVs, released by a wide range of cells, including platelets, monocytes, neutrophils, endothelial, and epithelial cells, serve as a significant source of both procoagulant and anticoagulant factors and can directly participate in the initiation and propagation of the coagulation cascade [66]. Functionally, the ability of sEVs to either promote or inhibit blood clot formation is largely determined by their cellular origin and the specific membrane receptors they express [67]. Small EVs support coagulation primarily through the externalization of phosphatidylserine (PS), a negatively charged phospholipid that provides a catalytic surface for the assembly of key coagulation complexes. This includes the tenase complex (factors VIIIa, IXa, and X) and the prothrombinase complex (factors Va, Xa, and II), both essential for the progression of the coagulation cascade. Additionally, PS may facilitate the conversion of tissue factor (TF) from its encrypted, inactive form into a biologically active state, further enhancing the procoagulant potential of sEVs [67]. TF is a transmembrane glycoprotein of approximately 47 kDa and serves as the primary initiator of the extrinsic coagulation pathway. Upon binding to Factor VII, TF forms the TF–FVIIa complex, which subsequently activates Factor X—central to the extrinsic pathway—and, to a lesser extent, Factor IX, which is part of the intrinsic pathway. These factors are key physiological components in the initiation and amplification of the coagulation cascade [68]. The co-expression of PS and TF on sEVs provides both a catalytic surface and an enzymatic trigger, making them highly procoagulant. Platelet-derived EVs are the most abundant subtype in circulation and play important roles in both physiological hemostasis and pathological coagulopathies. In addition, endothelial cells, monocytes/leukocytes, erythrocytes, and cancer cells are recognized sources of sEVs with strong procoagulant potential. For example, platelets release a large number of sEVs at the time of activation or injury [69] (Figure 4).

Disseminated intravascular coagulation (DIC) is an acquired syndrome marked by widespread activation of the coagulation cascade, leading to the formation of microthrombi and often resulting in the consumption of clotting factors and platelets. Small EVs can significantly amplify this pathological response through the expression of procoagulant molecules, contributing to both the initiation and propagation of DIC. This makes sEVs not only valuable biomarkers but also active mediators in the development of acute and chronic coagulopathies [70]. Recent findings have underscored the pivotal roles of tissue factor procoagulant activity (TF-PCA) and TFPI during the early stages of sepsis-induced DIC. Importantly, only sEVs-associated and functionally active TF, not merely TF antigen levels, demonstrated predictive potential for DIC onset. These insights suggest that targeting sEVs-associated TF activity could enhance diagnostic precision and inform therapeutic strategies aimed at mitigating the severe consequences of DIC in septic patients [70]. In a clinical study by Park et al. [71], the link between sepsis and coagulopathies was investigated through proteomic analysis of plasma-derived sEVs. The authors demonstrated that septic patients exhibit a significantly increased number of circulating sEVs, which display a markedly altered protein profile compared to healthy controls. Among the identified proteins, many are involved in key coagulation and thrombogenic pathways, such as von Willebrand factor and several components of the complement cascade. These findings suggest that sEVs not only reflect the hypercoagulable state of septic patients but may also actively participate in coagulation activation, contributing to the development of DIC. The observation that many of these proteins are not detectable in the free plasma proteome further supports the notion that sEVs represent a distinct, functional, and potentially pathogenic microenvironment [71]. Another experimental study by Lin et al. [72] explored the role of brain-derived sEVs as active mediators in the pathogenesis of DIC during sepsis. Using a rat model of sepsis, the authors observed that the brain rapidly releases large quantities of sEVs into the systemic circulation. These plasma vesicles, identified by specific neuronal markers, exhibited a pronounced procoagulant phenotype by exposing PS and facilitating platelet interaction, with a preferential activation of the extrinsic pathway mediated by TF. This procoagulant activity resulted in a hemostatic profile characteristic of DIC, as evidenced by increased plasma levels of thrombin-antithrombin complexes, D-dimer, and PAI-1, along with a concurrent reduction in fibrinogen [72].

Other clinical observations support the idea that neutrophil-derived sEVs, through their miRNA cargo, may actively participate in the development of sepsis-induced coagulopathy and could serve as novel biomarkers or therapeutic targets for coagulopathies associated with sepsis [73]. A study by Ye et al. [73] focused on profiling circulating miRNAs within neutrophil-derived sEVs in septic patients affected by coagulopathy. The researchers identified miR-150-5p, a crucial modulator of cellular defense mechanisms and apoptosis, as significantly decreased in sEVs of septic patients compared to healthy individuals, suggesting that the downregulation of this miRNA might worsen the clinical condition by exacerbating inflammatory processes [73].

Small EVs actively contribute to the thrombo-inflammatory coagulopathy observed in many COVID-19 patients, particularly in advanced or critical stages of the disease. A study by Campello et al. [74] confirmed elevated levels of sEVs originating from platelets (P-Selectin^+^ sEVs) and leukocytes (CD45^+^ sEVs), which persisted beyond the acute phase of the disease and correlated with the severity and persistence of symptoms. For example, P-Selectin^+^-EV concentrations exceeding 1054/µL were associated with an increased risk of thrombosis [74]. Further clinical studies have demonstrated that elevated levels of sEVs are associated with an increased risk of thrombotic events and reduced survival in patients with COVID-19. In comparative analyses, patients with severe COVID-19 were evaluated alongside those with moderate disease and individuals with non-COVID-19-related septic shock. Compared to the septic shock group, COVID-19 patients exhibited a distinct coagulopathy profile, characterized by significantly higher levels of sEVs-associated TF and enhanced EVs-mediated fibrinolytic activity. Changes in circulating platelet-derived sEVs subpopulations and distinct sEVs signatures were identified and were associated with coagulation activity. Interestingly, these sEVs offered additional prognostic value to D-dimer levels during the progression of COVID-19, suggesting that molecular signatures of sEVs may enable the early identification of critically ill patients, potentially before conventional coagulation parameters, such as elevated D-dimer levels, become apparent [75].

Although the role of EVs in sepsis and multi-organ failure is a topic of growing interest, the literature still lacks a definitive analysis comparing the relative contributions of lEVs and sEVs. Large EVs, due to their size and content of PS and TF, are more strongly associated with procoagulant, endothelial, and microvascular injury in the acute phase [76], whereas sEVs also act as potent modulators of immune signaling and remote organ inflammation, contributing particularly to intercellular communication [45]. Notably, clinical studies have reported that higher levels of PS-containing sEVs in the plasma of septic patients tended to be associated with a lower risk of mortality and multi-organ failure, suggesting a potential protective role in specific contexts [77].

Alongside these potent procoagulant features, lEVs may also carry anticoagulant components such as tissue factor pathway inhibitor (TFPI), thrombomodulin, or endothelial protein C receptor, which can partially counterbalance their thrombin-generating capacity and modulate the overall hemostatic impact. The predominant TF-dependent activity of lEVs has also been observed when their contribution to procoagulant activity was evaluated in thrombo-inflammatory diseases such as SARS-CoV-2 infection. In this setting, a clinical study demonstrated that COVID-19 is associated with a major shift in the hemostatic balance toward a procoagulant phenotype, supported by increased levels of TF-positive platelets and lEVs [78]. Also, in thrombo-inflammatory conditions such as sepsis or SARS-CoV-2 infection, the enhanced procoagulant potential that characterizes the infection is predominantly supported by lEVs, although both TF-positive sEVs and lEVs increase during the acute phase of the disease and return to normal levels with infection remission [79]. Therefore, circulating lEVs may also represent a promising target for future strategies aiming at reducing coagulopathies.

## 6. Role of sEVs in Organ Failure

Organ failure, affecting the heart, lungs, and kidneys, is a frequent and critical complication in sepsis [1,2]. Clinical studies have reported that elevated plasma levels sEVs can serve as biomarkers to identify severity of organ failure and are predictive of mortality in patients with sepsis [80]. In the context of acute lung injury, Mahida et al. [81] determined the phenotype of sEVs in the bronchoalveolar lavage fluid of patients with ARDS. The authors showed that patients with sepsis-related ARDS had a significantly higher number of CD14^+^ and CD81^+^ (CD14^+^/CD81^+^) sEVs, resulting from monocytes, than patients with sepsis without ARDS. The number of CD14^+^/CD81^+^ sEVs in bronchoalveolar lavage fluid correlated with increased mortality in patients with ARDS. This suggests that a high CD14^+^/CD81^+^ sEVs count found in patients’ body fluids could be a potential biomarker for the severity of disease and mortality in sepsis-related ARDS [81]. Other experimental studies have suggested a pathophysiological role of sEVs in lung injury. In a sepsis model of CLP, transfer of sEVs isolated from the blood of septic rats to healthy rats induced lung damage similar to that observed in septic mice [82]. The acute lung injury was associated with an increase in plasma levels of factor TNF-α, IL-6, and HMGB1, suggesting a systemic inflammatory response induced by administration of septic sEVs. These results support the hypothesis that, in severe sepsis conditions, sEVs can acquire pro-inflammatory and pro-thrombotic properties, actively contributing to the establishment of organ damage [82]. A recent study [83] further supported that the pathogenic role of sEVs in sepsis may be related to specific cargo content. In an in vitro cellular system of human lung epithelial cells, sEVs isolated from neutrophils of patients with sepsis induced inhibition of cell proliferation and activation of pyroptosis through the NOD-like receptor family pyrin domain containing 3/Toll-like receptor 4 (NLRP3/TLR4) pathway. Additional mechanistic analysis revealed that the protein S100A8 was highly expressed in the neutrophil-derived sEVs and was in part responsible for the cell injury [83]. The idea that sEVs play a dual role, both as mediators of disease processes and as normal components involved in maintaining cellular balance, has been investigated in a recent clinical study in patients with ARDS [84]. In this study, the investigator identified a distinct profile of miRNAs carried in circulating sEVs of patients with sepsis-related ARDS in comparison to septic patients without ARDS, suggesting a direct role of these RNAs in the progression of lung damage. The investigators also associated these changes with an increased tendency for thrombosis formation, concluding that in cases of sepsis with respiratory involvement, sEVs may actively contribute to lung injury.

Acute cardiac damage, such as a myocardial infarction or cardiogenic shock, reduces the heart’s ability to pump blood, leading to ischemia and multiple organ failure, particularly during the worsening of sepsis [85]. The contribution of sEVs in modulating myocardial injury has been proposed in several experimental and clinical studies. For example, a recent study reported that sEVs isolated from the serum of septic patients could promote apoptosis and inhibit aerobic glycolysis when incubated in vitro with human AC16 cardiomyocytes. These effects were attributed to the upregulation of miR-1262 in the sEVs cargo [86]. Endothelial-derived sEVs miR-126 has also been shown to have a cardioprotective effect in a murine model of experimental sepsis. Administration of sEVs enriched with miR-126 in mice with sepsis-induced cardiomyopathy improved cardiac performance by downregulating adhesion molecules and attenuating immune cell infiltration [87]. These findings underscore the complex role of sEVs, which can regulate tissue repair and ameliorate organ function depending on their cell of origin and their cargo content. These findings also suggest that specific sEVs miRNAs play a critical role in septic cardiac dysfunction and may represent a novel therapeutic target. During sepsis-related myocardial dysfunction, sEVs may be released by the affected cardiomyocytes. A recent experimental study in septic rats [88] demonstrated that cardiomyocytes release sEVs containing mitochondrial fragments and p62. Notably, the release of sEVs was not a passive consequence of cellular stress but rather an active mechanism of inflammatory propagation, driven by mitochondrial stress and autophagy. A recent study by Hegyesi et al. [89] suggests that circulating sEVs may also represent biomarkers of sepsis-induced cardiomyopathy. Using a mouse model of LPS-induced systemic inflammatory response syndrome, the authors showed increased plasma levels of cardiomyocyte-derived small and medium EVs enriched with troponin I and muscle-associated glycogen phosphorylase.

The involvement of sEVs in lung injury and cardiac dysfunction represents just one facet of their broader contribution to sepsis-induced organ failure. These particles have also been implicated in the pathogenesis of acute kidney injury (AKI), where they contribute to endothelial dysfunction, tubular damage, and immune activation, further amplifying the cascade of multi-organ dysfunction. For instance, sEVs derived from human endothelial progenitor cells (EPC) enriched with miR-93-5p have demonstrated the ability to attenuate sepsis-induced AKI by suppressing inflammation and apoptosis through epigenetic regulation in a murine model of CLP [90]. The specific cargo of platelet-derived sEVs has also been correlated to sepsis-related AKI. For example, the level of miR-223-3p in the platelet sEVs isolated from the serum of patients with sepsis was significantly lower than that of the healthy controls. The level of miR-223-3p was also decreased in the platelet sEVs of a mouse model with sepsis-induced acute renal injury [91]. Functionally, miR-223-3p carried by sEVs was shown to target NLRP3, a key component of the inflammasome complex, which promotes pyroptosis and amplifies the inflammatory response. In the context of AKI, sEVs released into the urine may represent a valid biomarker. A clinical study by Panich et al. [92] identified Activating Transcription Factor 3 (ATF3) as a potential early marker of sepsis-AKI detected within urinary sEVs collected from a cohort of patients with sepsis-AKI compared to those with sepsis without AKI and healthy controls. Notably, ATF3 is known to exert anti-inflammatory and anti-apoptotic effects by modulating transcriptional programs within renal tubular cells. Therefore, the presence of ATF3 within sEVs may suggest that these particles are released to contribute to renal protection [92].

## 7. Role of sEVs in Burn-Related Sepsis

Severe burn injuries cause extensive tissue damage and compromise the skin barrier, making the body highly susceptible to infections. This creates a favorable environment for microbial invasion and systemic spread of pathogens. The combination of burn injury and sepsis is associated with high mortality and requires urgent, intensive medical care. It poses a clinical challenge in controlling infection, managing inflammation, and maintaining hemodynamic stability [93].

In a single-center study, elevated plasma levels of leukocyte-derived sEVs in burn victims with sepsis were related to higher mortality rates [94]. In another clinical observational study, Yang and colleagues [95] reported that burn injury induced a significant increase in circulating sEVs, with levels peaking at the time of admission and subsequently declining during the recovery phase. When the cellular origin of these vesicles was investigated, the authors observed that sEVs were released by leukocytes and endothelial cells. Importantly, the sEVs exhibited pro-inflammatory activity when tested in vitro in endothelial cells, as these particles were able to induce a decrease in transcellular barrier resistance and loss of cell–cell junction continuity, thus supporting an important role of sEVs in endothelial dysfunction. The pro- and anti-inflammatory functions of sEVs was also investigated by Willis et al. [96], in which sEVs were isolated from the plasma of patients with burn injuries at early (<72 h) and late (>14 days) post-injury time points. These vesicles were applied to cultures of human monocytic cells in the presence of LPS to mimic the immune response in sepsis. The investigators observed that both early and late burn-derived sEVs significantly increased the secretion of IL-6 in LPS-stimulated monocytes compared to sEVs from healthy controls. However, the most distinctive finding was that only the sEVs collected late after burn injury induced a significant increase in IL-10 secretion. This shift toward an IL-10/IL-6 cytokine profile suggests a reprogramming of macrophage responses toward an immunosuppressive phenotype, a pattern that has been previously associated with poor clinical outcomes in burn patients and is characteristic of the immune-paralysis phase observed in sepsis [96].

Small EVs may represent a sensitive and specific biomarker for sepsis in the vulnerable population of burn victims. In a single-center clinical study, Schiavello and colleagues [97] focused on the molecular dynamics of the sEVs miRNA content and the occurrence of sepsis complications in burn patients. Comparative analyses revealed that miR-34a-3p and miR-193a-5p positively correlated with the severity of sepsis as determined by the Sequential Organ Failure Assessment index [97]. Following a burn injury, early diagnosis of sepsis is a significant clinical challenge due to the overlapping symptoms of systemic inflammation and infection. In this context, a study conducted by O’Toole et al. [98] proposed an innovative approach based on the analysis of plasma sEVs of burn patients using Raman spectroscopy as a rapid and non-invasive method. The results showed that sEVs were more abundant in patients with sepsis after burn injury, both in terms of concentration and distinct chemical signatures detected spectrally. This difference was attributed in part to the presence of bacterial sEVs, released into the bloodstream during infection. The discriminant analysis allowed researchers to distinguish septic from non-septic patients with high accuracy, further supporting the role of sEVs as a biomarker for sepsis in the burn population [98]. This approach opens new perspectives for early and personalized diagnosis, potentially improving the prognosis of burn patients at risk of infectious complications.

## 8. Standard and Novel Methods of sEVs Extraction and Isolation

The investigation of sEV characteristics is essential to improve our understanding of their biological role and their potential use for therapeutic and diagnostic applications. A critical step in characterizing sEVs is their isolation. A variety of methods are employed, each having benefits as well as disadvantages. Although the ideal separation method should be fast, reliable, and affordable, the nanoscale size of these particles necessitates a multi-step method. Most extraction methods rely on strategies based on sEVs size, density, surface markers, and other physical and biological properties.

The methods can be classified into three main categories: precipitation-based, affinity-based, and chromatography-based approaches. The most widely used and conventional method, ultracentrifugation, is often considered the gold standard [99]. It separates particles based on size and density through a series of centrifugations at progressively increasing speeds. Initial centrifugation at low speed removes cells and debris, followed by intermediate-speed spins to isolate apoptotic bodies and microvesicles, and finally, high speed ultracentrifugation (over 100,000× *g*) to isolate sEVs [100]. While effective, this method is labor-intensive, time-consuming, and prone to contamination by other similar-sized particles [100]. Polymer-based precipitation, using agents like polyethylene glycol (PEG), aggregates and precipitates sEVs from biological fluids. This approach is faster than ultracentrifugation but generally suffers from lower specificity and greater contamination with soluble materials [101]. Immunoaffinity capture methods use antibodies targeting specific sEVs surface markers, such as CD63, CD9, and CD81. This method provides high purity and enrichment of sEVs from specific subpopulations. However, if vesicles lack the targeted marker, they may go undetected, limiting comprehensive sEVs profiling [102]. Size Exclusion Chromatography (SEC) is widely recognized as one of the most effective techniques for purifying sEVs [103]. The SEC extraction system avoids chemical interactions between the vesicles and the separation matrix, preserving structural and biological integrity. The process separates particles based on particle size: larger particles, such as sEVs, elute first, while smaller molecules are retained longer as they pass through the porous structure. SEC is compatible with many biological fluids, including plasma, serum, cerebrospinal fluid, and cell culture media, is reproducible, scalable, and cost-effective, making it suitable even in resource-limited settings. Compared to ultracentrifugation, SEC often yields sEVs with higher recovery rates and improved purity [103,104]. Hybrid approaches, such as coupling ultracentrifugation with SEC, are used to increase recovery efficiency and purity without requiring molecular labeling [105]. Electrostatic interaction-based methods allow selective sEV isolation by exploiting lipid membrane properties. For example, MagCapture beads, which use Tim4 protein conjugated to magnetic particles, bind PS-positive sEVs in a calcium-dependent manner [106]. This method enables high purity during the extraction of sEVs with minimal contamination, suitable for sensitive applications like electron microscopy [106].

In addition to conventional methods, commercial kits have been developed to extract sEVs from biological fluids like serum. These kits provide fast, standardized, often ultracentrifugation-independent protocols and support scalable, reproducible workflows. Commonly used kits include exoEasy, ExoQuick, Exo-spin, ME kit, ExoQuick Plus and Exo-Flow, which exploit different mechanisms such as polymeric precipitation (ExoQuick, Total Exosome Isolation), specific affinity for surface markers or PS (MagCapture, Exo-Flow, PureExo, ME kit), spin column affine membranes (exoEasy) and dimensional exclusion chromatography (qEVoriginal, Exo-spin) [107]. Each method offers a different balance between yield, purity, and operational complexity. Depending on the starting sample, different strategies may be more appropriate.

Electrochemical analysis of sEVs surface charge and dielectric properties represents an emerging approach with diagnostic and therapeutic potential. Small EVs typically exhibit a negative surface charge, due to phospholipids and membrane proteins. This can be quantified by ζ-potential, which measures their interaction with all positively charged particles [108]. Zhang and coworkers [109] developed a novel frequency-dependent impedance testing technique using electrical impedance spectroscopy (EIS) to characterize sEVs by their dielectric properties. Their dielectrophoretic biosensor identifies sEVs clusters from various cellular sources and connects their impedance signals to distinct cytoplasmic and membrane characteristics. These findings suggest that portable EIS-based devices may provide real-time bedside detection of sEVs biophysical and biochemical properties, supporting clinical applications [109].

Other recent innovations such as microfluidic-based isolation have emerged as promising alternatives for achieving scalable, high-purity vesicle recovery [110]. These platforms can exploit physical mechanisms like deterministic lateral displacement, inertial focusing, viscoelastic flow, or nano-filtration, to discriminate vesicles by size or density or apply chemical strategies (such as immunocapture on functionalized surfaces or magnetic beads) to achieve molecularly selective isolation. By integrating multiple operations into a chip, microfluidic systems reduce sample and reagent requirements, shorten processing times, and maintain sEVs integrity, while offering compatibility with downstream molecular and functional assays. These chips could potentially facilitate point-of-care diagnostic tools for clinical applications.

Another promising technology of sEV isolation is acoustic trapping. This label-free technique uses ultrasonic standing waves and isolates the vesicles from their surrounding fluid based on differences in their acoustic properties, while preserving their structural and biochemical integrity [111]. Recent developments, such as the Multinode Acoustic Trap platform, have enhanced capture efficiency, scalability, and throughput, positioning acoustic trapping as a viable method for high purity sEV isolation in clinical research settings [112].

The identification of the cellular origin of circulating sEVs remains a crucial yet challenging step toward the discovery of novel biomarkers and the implementation of personalized therapeutic strategies. While conventional flow cytometry is routinely applied to large EVs using minimal plasma volumes, the resolution of older instruments is generally insufficient to detect particles smaller than 300 nm. Recent advancements in high-sensitivity flow cytometry, including nano-flow cytometry, have enabled the detection and phenotyping of individual sEVs as small as 40 nm, significantly enhancing analytical resolution [113]. Nonetheless, potential pitfalls in sEVs flow cytometry such as coincidence, swarm detection, and antibody- or lipoprotein-mediated background artifacts are frequently reported [114]. Complementary approaches, including immunoaffinity capture using cell-type-specific surface markers (e.g., CD41 for platelets, CD31 for endothelial cells, CD45 for leukocytes), single-vesicle imaging via super-resolution fluorescence microscopy or high-sensitivity imaging flow cytometry, and label-free molecular profiling through Raman spectroscopy, lipidomics, and transcriptomics, offer additional strategies to determine cellular origin of vesicles [115,116]. Together, these complementary methodologies provide an increasingly robust toolkit for defining the cellular source of sEVs, an indispensable step toward their translation into targeted, patient-tailored therapies.

## 9. Modern Systems of sEV-Based Drug Delivery and Experimental Therapy

Small EVs are also increasingly gaining attention in the international scientific community as innovative tools for drug delivery. Their intrinsic properties make them particularly well-suited for applications in precision medicine. Compared to traditional delivery systems such as liposomes or synthetic nanoparticles, sEVs offer several notable advantages. Owing to their natural biogenesis, they exhibit high biocompatibility and low immunogenicity, reducing the risk of adverse immune responses. Moreover, sEVs demonstrate remarkable stability in circulation and, due to their lipid-based structure, are capable of efficiently crossing biological membranes, including complex barriers such as the blood–brain barrier. They also exhibit tissue tropism, enhancing their potential for targeted delivery. These features collectively position sEVs as a superior and versatile system of drug delivery [117].

There are two main strategies for loading therapeutic cargo into sEVs: endogenous and exogenous loading. In the endogenous approach, the parental cells are genetically modified or pharmacologically stimulated to incorporate specific molecules into sEVs during their natural biogenesis. This method offers several advantages, including preserved membrane integrity and high cargo selectivity, as the parent cells can be engineered to express specific ligands or receptors. For example, in a recent study, neutrophil membrane-engineered sEVs were prepared from the root of *Panax ginseng* and were loaded with miRNA-182-5p to treat mice with LPS-induced acute lung injury [118]. Treatment with these engineered particles ameliorated lung injury and reduced infiltration of inflammatory cells by targeting the NLRP3 pathway [118]. However, endogenous loading typically requires prolonged cell culture periods and may not be suitable for certain drugs, particularly those that are cytotoxic or unstable under physiological conditions [119].

In contrast, the exogenous approach involves introducing the therapeutic cargo into already isolated sEVs using physical or chemical methods (e.g., electroporation, sonication, or incubation) [120]. This strategy offers greater flexibility, shorter processing times, and compatibility with a broader range of molecules. An example is the use of engineered-sEVs loaded with a modified form of IκBα, called super-repressor IκBα (srIκBα), which blocks upstream activation of the transcription factor NF-κB, thereby preventing the propagation of subsequent pro-inflammatory signals (TNF-α, IL-1β, and IL-6) [117]. In mouse models of LPS- or CLP-induced sepsis, systemic administration of sEVs enriched with srIκBα significantly reduced mortality, attenuated the cytokine storm, and protected organs from histological damage, particularly the kidneys. The sEVs were rapidly taken up by neutrophils and macrophages in the liver, spleen, and kidneys, demonstrating effective targeting capability. In vitro, the same treatment inhibited NF-κB activation in mononuclear and endothelial cells by reducing adhesion molecule and chemokine expression, thereby limiting leukocyte recruitment [117]. Nevertheless, this method carries the risk of compromising vesicle integrity and contaminating the preparation with exogenous DNA or RNA, potentially affecting biological functionality and interfering with intended therapeutic activity [121,122].

Despite the promise of sEV-based delivery systems, several limitations must be considered. A key challenge is the low production yield of sEVs due to limited natural release by cells and loss of vesicles during isolation or cargo-loading procedures. It is estimated that a single therapeutic dose may require approximately 10^13^ sEVs per patient [123], highlighting the need for scalable production methods. To address this bottleneck, various strategies have been proposed, including electrical stimulation, biological transfection, and three-dimensional cell culture systems. Three-dimensional cell culture systems, supplemented with specific nutrients and growth factors, have been shown to increase sEVs yield by up to 100-fold compared to conventional two-dimensional cultures [124].

Several clinical trials (phase III and IV) are currently underway to evaluate sEVs in human therapy, most commonly in COVID or ARDS patients. These studies employ sEVs derived from stem cells or engineered sources, showing encouraging signs in terms of safety and stability [125]. Experimental studies in mice demonstrated that MSC-derived sEVs have demonstrated regenerative potential by delivering growth factors and RNA that stimulate cell proliferation and differentiation, thereby supporting tissue healing and repair. These vesicles also show promise in complex conditions such as sepsis [126,127]. For example, an experimental study in mice by Wang et al. proposed the use of adipose-derived MSC sEVs as a therapeutic approach for sepsis and its complications, including lung injury [126]. These sEVs suppressed inflammatory cytokine production, particularly IL-27 in macrophages, resulting in reduced macrophage infiltration, lowered cytokine levels (IL-6, TNF-α, and IL-1β), and improved pulmonary histopathology, including reduced edema, vascular permeability, and tissue damage. These findings highlight the potential of MSC-derived sEVs as a cell-free therapeutic strategy for sepsis, due to their ability to modulate the immune response and attenuate inflammation in a precise and effective manner [126]. Similarly, Li et al. [127] proposes the use of sEVs derived from bone marrow–MSCs as a potential cell-free therapeutic strategy for the treatment of ARDS associated with sepsis. In a murine model of CLP-induced sepsis, intratracheal administration of MSC-derived sEVs led to a reduction in pulmonary histological damage and suppressed HMGB1 and IL-6 in bronchoalveolar lavage fluid, indicators of attenuated inflammation and tissue injury. These findings strongly support the potential of MSC-derived sEVs as cell-free therapies with advantages over traditional cell therapies in terms of safety, delivery, and biological regulation [127].

Recent advancements in sEV-based therapies for tissue repair include incorporating sEVs into biomaterial scaffolds, such as hydrogels, for improved therapeutic retention, which has been tested in infected wounds [128]. Because of their porous structure, biocompatibility, and ability to mimic the extracellular matrix, hydrogels provide a protective environment for sEVs and enable controlled, sustained release. Additionally, they can be engineered to respond to specific stimuli in the wound microenvironment, such as pH or oxidative stress, further enhancing therapeutic efficacy. Small EV-loaded hydrogels have demonstrated improved retention and stability of sEVs at the site of infected wounds, addressing the challenge of rapid clearance or degradation of sEVs in vivo. These hydrogels serve as a localized delivery system, gradually releasing sEVs to sustain their bioactive effects over time facilitating wound healing through their ability to deliver pro-angiogenic factors, which play pivotal roles in vascular endothelial regeneration [129]. The combined use of sEVs and hydrogels has been shown to accelerate healing in mouse models of wound healing by promoting collagen deposition, new blood vessel formation, hair follicle regeneration, and even nerve repair [130]. Hydrogels have been engineered to release sEVs sequentially or to incorporate additional bioactive agents, such as growth factors or antibacterial nanoparticles, thereby expanding and enhancing therapeutic possibilities. An emerging direction is 3D bioprinting, which enables precise fabrication of custom scaffolds containing EVs and hydrogels. This technology allows for patient-specific wound shaping, enhanced sEVs distribution, and integration of advanced properties such as electrical conductivity or antioxidant properties [130].

## 10. Future Directions for Bridging the Bench to Bedside

**Figure 5:**
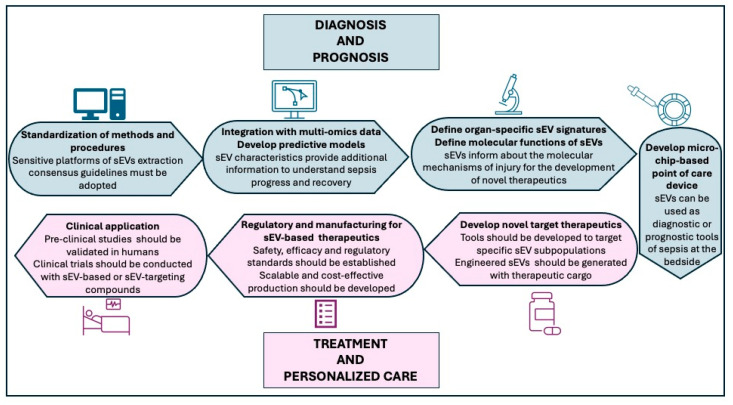
Strategic directions for advancing the clinical application of sEVs in sepsis. The roadmap highlights seven major areas: (1) Standardization of sEV isolation and characterization, emphasizing the need for consensus protocols to improve reproducibility across studies; (2) Integration with multi-omics, enabling biomarker discovery and predictive modeling for personalized care; Clinical validation, underscoring the importance of robust clinical trials to confirm diagnostic and therapeutic potential; (3) Organ-specific sEV profiling, to deepen understanding of organ dysfunction and support targeted interventions; (4) Point-of-care technologies, such as microchip-based platforms for rapid bedside detection of sEVs, facilitating early diagnosis and timely treatment; (5) Development of therapeutic products targeting sEVs, including engineered sEVs; (6) Regulatory and manufacturing frameworks, focusing on safety, efficacy, and scalable production methods; and (7) Clinical application, emphasizing the need for well-designed clinical trials to confirm diagnostic and therapeutic utility.

Despite significant progress in understanding the role of sEVs in sepsis, several challenges remain that must be addressed to facilitate clinical translation (Figure 5). First, standardization of EV isolation and characterization techniques is needed. The lack of uniform protocols contributes to variability in findings and limits cross-study comparisons. Adoption of consensus guidelines, such as those proposed by the ISEV is important to improve reproducibility. Second, while preclinical studies have demonstrated promising diagnostic and therapeutic applications of sEVs, clinical validation remains limited. Clinical trials will be essential to establish the utility of sEVs in real-world settings. Third, regulatory and manufacturing challenges must be addressed, including defining safety and efficacy standards for EV-based products, and developing scalable, cost-effective production methods suitable for clinical use. Fourth, the integration of sEV data with multi-omics approaches offers potential for biomarker discovery and for the development of predictive models for personalized care. Also, further research into organ-specific sEV signatures may enhance our understanding of organ dysfunction in sepsis and enable targeted interventions. Additionally, the use of microchip-based point-of-care devices presents a promising avenue for the rapid and sensitive detection of sEVs in clinical settings. These platforms can enable real-time monitoring of sepsis biomarkers at the bedside, facilitating early diagnosis and timely therapeutic interventions. By addressing these challenges, future research can help bridging the bench-to-bedside gap and pave the way for sEVs to become integral tools in the diagnosis, prognosis, and treatment of sepsis.

## 11. Conclusions

**Figure 6:**
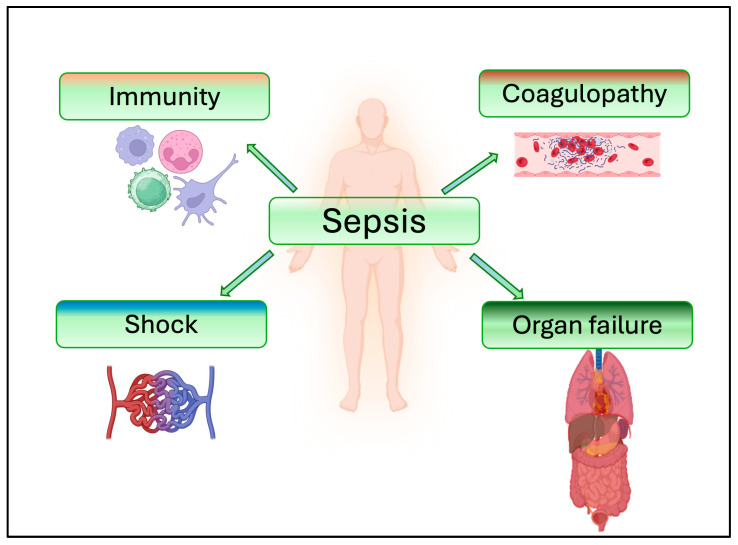
Involvement of extracellular vesicles in the progression of sepsis. This schematic illustrates the multifaceted involvement of small extracellular vesicles (sEVs) in the progression of sepsis. During sepsis, subpopulations of sEVs released from immune cells, endothelial cells, platelets, and damaged tissues can acquire specific cargo and contribute to key pathological processes. *Immunity:* the cargo of sEVs can amplify immune responses and promote systemic inflammation. Small EVs also influence immune cell activation, polarization, and apoptosis. *Coagulopathy:* Small EVs express both pro- and anti-coagulant factors and contribute to septic coagulopathies. *Shock:* Small EVs can impair vascular tone and endothelial barrier integrity, leading to hypotension and capillary leakage, which are central features of septic shock. *Organ failure:* Small EVs mediate intercellular communication that exacerbates inflammation, oxidative stress, and apoptosis in vital organs contributing to multi-organ failure.

In conclusion, sEVs emerge as pivotal players in the intricate landscape of sepsis and related organ injury (Figure 6).

By influencing immune modulation, endothelial stability, metabolic balance, and coagulation pathways, sEVs not only reflect the systemic complexity of sepsis but also offer a biologically grounded platform for innovation in diagnostics and treatment. Small EVs are, in fact, not merely passive biomarkers or delivery vehicles, but dynamic regulators of intercellular communication, with the potential to reshape the cellular environment. As our understating of their role deepens, the successful translation of sEV-based strategies into clinical practice will depend on standardization of isolation and characterization methods, rigorous mechanistic studies, and the development of safe, targeted delivery systems.

As research progresses, sEVs may well become a cornerstone of next-generation therapies for sepsis and the related multiple organ failure, offering new hope not only for precision and efficacy in critical care medicine, but also as a proficient tool for early diagnosis, potentially enabling earlier clinical intervention and improved clinical outcomes. Nevertheless, significant challenges remain, including standardization of isolation techniques, regulatory hurdles, and cost-effectiveness, which must be addressed to enable successful clinical translation.

## Figures and Tables

**Table 1:** Characteristics of extracellular vesicles according to size, biogenesis and composition. Details about the technology of extraction are described in the original reference.

	Exosomes	Microvesicles	Apoptotic Bodies	References
**Size**	30–150 nm	100–1000 nm	1–5 μm	[3,4,5,6,7,8,9]
**Origin**	From multivesicular bodies	From plasma membrane	From plasma membrane and cellular fragment	[10,11]
**Mechanism of formation**	Released by fusion with plasma membrane	From swelling towards the outside of the plasma membrane	Apoptotic process byproducts	[12,13]
**Content**	Proteins, lipids, RNA, DNA, mRNA and miRNA	Proteins, lipids, RNA, DNA, mRNA and miRNA	Cytosol portions, degraded proteins, DNA fragments, or organelles	[14]
**Membrane markers**	CD9, CD63 and CD81, and ESCRT proteins Alix and TSG101	Selectins, flotillin-2, ARF6 and CD40	Caspase3, CD3 and CD44	[15]
**Role**	Cell–cell communication	Coagulation	Cell clearance	[16]
**Charge**	Negative	Negative	Not reported	[17,18]
**Shape at EM**	Small and spherical	Small and spherical	Heterogenous in size and shape	[19,20]

## Data Availability

No new data were created or analyzed in this study.
